# How Contextual Constraints Shape Midcareer High School Teachers' Stress Management and Use of Digital Support Tools: Qualitative Study

**DOI:** 10.2196/15416

**Published:** 2020-04-27

**Authors:** Julia B Manning, Ann Blandford, Julian Edbrooke-Childs, Paul Marshall

**Affiliations:** 1 University College London Interaction Centre London United Kingdom; 2 Institute of Healthcare Engineering University College London London United Kingdom; 3 Evidence-based Practice Unit Anna Freud Centre and University College London London United Kingdom; 4 Department of Computer Science University of Bristol London United Kingdom

**Keywords:** school teachers, stress, health, self-management, computers, technology, qualitative research, secondary schools, wearable devices, mobile applications, education

## Abstract

**Background:**

Persistent psychosocial stress is endemic in the modern workplace, including among midcareer high school (secondary comprehensive) teachers in England. Understanding contextual influences on teachers' self-management of stress along with their use of digital health technologies could provide important insights into creating more usable and accessible stress support interventions.

**Objective:**

The aim of this study was to investigate the constraints on stress management and prevention among teachers in the school environment and how this shapes the use of digitally enabled stress management tools.

**Methods:**

Semistructured interviews were conducted with 14 teachers from southern England. The interviews were analyzed using thematic analysis.

**Results:**

Teachers were unanimous in their recognition of workplace stress, describing physical (such as isolation and scheduling) and cultural (such as stigma and individualism) aspects in the workplace context, which influence their ability to manage stress. A total of 12 participants engaged with technology to self-manage their physical or psychological well-being, with more than half of the participants using consumer wearables, but Web-based or smartphone apps were rarely accessed in school. However, digital well-being interventions recommended by school leaders could potentially be trusted and adopted.

**Conclusions:**

The findings from this study bring together both the important cultural and physical contextual constraints on the ability of midcareer high school teachers to manage workplace stress. This study highlights correlates of stress and offers initial insight into how digital health interventions are currently being used to help with stress, both within and outside high schools. The findings add another step toward designing tailored digital stress support for teachers.

## Introduction

### Background

Teaching is one of the most stressful professions. Surveys have shown teaching to be in the top 6 (out of 26) professions that report the worst scores for physical health, psychological well-being, and job satisfaction [1]. Excessive, chronic stress is acknowledged as a condition of mental or emotional strain or tension, which can have psychological, behavioral, and physiological effects [2]. “Teacher stress” was first specifically described by Kyriacou [3] as “a response syndrome of negative effects (such as anger or depression) resulting from the general teacher’s job”. Teachers’ experiences of stress at work have received extensive coverage in the literature [4-8], with stress sources recently summarized as the following: (1) teaching unmotivated students, (2) maintaining discipline, (3) time pressures and workload, (4) coping with change, (5) being evaluated by others, (6) dealings with colleagues, (7) self‐esteem and status, (8) administration and management, (9) role conflict and ambiguity, and (10) poor working conditions [9]. Some of these sources reflect contextual factors that are intrinsic to the job and shape the method of teaching (eg, classroom setting, timetabling, and instructing), which have also been referred to as the *school climate* [10,11]. Stress coping strategies have been categorized as physiological (eg, relaxation, meditation, and aerobic activity), situational (eg, changing personal reaction or altering work environment), or cognitive (eg, controlling emotions, problem solving, or time management) [12]. Cognitive strategies have been shown to be used more than situational strategies [13,14], but physiological interventions, including mindfulness, have been growing in popularity [8,15,16].

Stress management has often been analyzed at the level of intervention. Stress interventions for teachers have been categorized by the level of mediation: (1) organizational, focused on the organization’s culture; (2) organization-individual interface, including building workplace relations; or (3) individual, where people are taught practices to manage stress [17]. The following fourth intervention categorization has subsequently been added: classroom level, influencing student functioning and behavior [18]. Studies to date indicate that organizational health interventions have the most potential to reduce general teachers’ work-related stress [19]. Yet, this seems to have only been verified in the US primary (elementary) school system, not in other countries (eg, England) and never in high schools. In addition, although some organizational contextual factors such as school leadership, workplace culture, and teacher autonomy have been identified as playing a critical role in managing stress, it is noted that they are in the purview of *schools and employing bodies* and not under the control of an individual’s authority or management [20].

A systematic review of studies on the effectiveness of taught interventions aimed at reducing general teacher burnout (ie, stress-induced work absence) found that in the 11 out of 23 controlled studies which included high school–level educators, cognitive behavioral therapy (CBT), mindfulness, and social support were the interventions that showed an effect, albeit small [21]. The lack of tailoring of these interventions to teachers’ work and context is suggested as the reason for the effect size being small, as tailored interventions showed more positive results. Tailoring included reflecting classroom situations and teacher-student relationships [22-24]. Many approaches to well-being, described as *whole school* do not include the staff’s own well-being [25-27], but there is an established literature on the link between teachers’ well-being and work-related outcomes [28-30], which some schools are beginning to recognize [31,32].

Technology has enabled stress reduction techniques to become digitized with digital health interventions (DHIs), generally known as electronic health (eHealth), and arguably more accessible, self-administered, and connected [33]. There is a growing body of studies detailing DHI use in the workplace. A meta-analysis of 21 randomized controlled trials (RCTs) on psychological interventions in the workplace utilizing Web-based programs (of which 3 used mobile apps) has shown improvements in employees’ well-being and work effectiveness [34]. In another review of 23 eHealth RCTs, improvements have been seen in mental health and stress symptoms [35], although it was observed that similar to nondigital approaches, the best outcomes depend on providing the right type of intervention to the correct target population. Interestingly, introducing activity tracking or tools (eg, exercise videos, Nintendo Wii) intended to increase exercise levels among nurses did not result in lifestyle behavior change, but it did serve as a stress reliever [36]. Wearable devices have received little attention in the workplace so far. One study on activity with a small sample found that among office workers, wearable feedback did not affect sitting time and that there were issues with accuracy, battery life, and real-time feedback [37]. Another study with office workers found that on applying machine learning to the collected heart and activity data, it was possible to automate a degree of mood recognition, including stress, and therefore make some objective predictions, which posited that this could enable the employer to change the work environment accordingly [38]. However, the accuracy of wearables in stress detection in real-world situations remains as issue, as does the lack of agreement on a standard stress measurement method [39,40]. Only one tailored eHealth stress reduction study by Ebert et al in Germany [41] appears to have been conducted in education: an internet-based Problem Solving Therapy (PST, a form of CBT) adapted for teachers. Averaging 19 years of teaching experience, participants had elevated symptoms of depression. The PST resulted in a reduction in their perceived stress but it is unclear whether the study resulted in any systematic implementation. In the literature, there is little evidence of the adoption of DHIs for teachers’ stress management in schools.

Teachers in the middle of their careers are at stages three and four in Huberman’s widely accepted 5 stages of the teacher career development model. This includes those with 7 to 30 years of practice with roles including coordinators, heads of department, and in personal development [42]. They are more likely to be under pressure because of the demands both in and outside the job [43], but as *survivors* of the early years, they should also have management strategies in place [44]. A 2012 German study of nearly 2000 teachers found that midcareer teachers have more tasks and invest more effort than colleagues who are at the beginning or end of their careers [45]. Therefore, the combination of pressure, adaption, and effort seen in senior teachers means it is appropriate to focus the investigation on their stress management, particularly in schools where there is already an acknowledgment of the philosophies of workplace health [46]. Tailoring technology to real-world situations and teachers’ existing behavior needs to be understood if we intend to align digital support in a meaningful way [34,35]. The literature on teacher stress is extensive but not conclusive.

### Objectives

There are gaps in understanding how the educational context influences the strategies that midcareer high school teachers are actually able to use to manage stress. Similarly, we do not know to what extent teachers have already adopted technology as a stress intervention or prevention tool and what the opportunities are for expansion.

A more nuanced understanding of these teachers’ real-world situation is required if we are to facilitate techniques for the reduction of stress, whether at the individual or organizational level. How does the school organizational context constrain individual teachers’ stress management strategies? Therefore, the aims of this study were to engage with midcareer high school (secondary comprehensive) teachers to understand (1) how workplace context influences stress management and (2) how this affects the current use of digital health technology, to ascertain the potential for technology support for stress reduction.

## Methods

### Study Design

The methodological orientation of this study was experiential qualitative research [47]. Exploring and understanding participants’ strategies in their everyday work context was desired so that these data might inform a future qualitative inquiry into the potential for integrating technological support for stress reduction among senior teachers.

### Teacher Recruitment

The initial selection of participants was purposive so that the target population of high school teachers in midcareer roles was secured. The inclusion criteria were to be a high school teacher in a management position, eg, in the senior leadership team, head of department or head of house, or a teacher who had previously held a senior role. Willingness to talk about stress was specified in the information sheet. No prior individual training in stress interventions was stipulated, but their school had to have demonstrated an awareness of the importance of well-being, such as through having a school lead on well-being. Staff from 5 different schools in the midlands, London, and southern England, who met the inclusion criteria, were recruited using the researcher’s networks (with the head teacher’s permission sought for 2 on-site sets of interviews). None of the participants had previously met the researcher. All volunteers were sent a comprehensive information sheet and consent form to read when they had indicated that they were willing to be interviewed. The consent and information forms specified the purpose of the interview, the interviewer’s department and university, and that the interviews would contribute to a study being undertaken as part of a PhD.

School-facilitated interviews were requested to take place during the school day to not cause teaching staff the inconvenience of having to stay on after hours. However, several staff members volunteered to be interviewed on a Friday afternoon after classes had finished. A printout of the information document was also given to participants at the start of the interview to ensure they had a physical copy to take away. This included information on where they could obtain free and confidential advice should they have felt in need of support after the interview had finished. This research was approved by the University College London (UCL) Research Ethics Committee approval ID number: UCLIC/1718/013/Staff Marshall/Manning.

### Interview Script Development

The interview guide was informed by the literature on stress in the school workplace as well as previous Education Support Partnership stress questionnaires [48] and Health and Safety Executive research [49,50]. The interview took a semistructured qualitative approach with questions devised to understand (1) how workplace context influences stress management and (2) how this affects the current use of digital health technology, to ascertain the potential for technology support for stress reduction.

The interview had been pilot tested with 2 teachers independently, an assistant head and a head of department from 2 separate schools. The data from these interviews were only used to refine the interview schedule and not used in the thematic analysis. The whole interview schedule is presented in Multimedia Appendix 1. For the purposes of this paper, the focus was on the influence of the workplace context and insights into the use of digital health tools. None of the interviews were terminated prematurely or repeated. They were timed to last for 30 min, with the shortest interview lasting 22 min and the longest interview lasting 43 min. 

### Data Analysis

All interviews were transcribed verbatim and analyzed using inductive thematic analysis. NVivo version 12 (QSR International Ltd) was used for the organization and development of codes and categories, and SimpleMind (version 1.22) was used for the mapping of relationships and themes by the researcher (JM). Transcripts were not returned to participants for comments or corrections (1) as the researcher did not want to add a time burden on the participant, (2) as the evidence is that it adds little to the accuracy of the transcript [51], and (3) to retain the responses as precisely as they had been provided at the time of the interview, when the interviewee had had the protected time and the safe space to respond spontaneously. No personal names or identifying remarks or subjects mentioned in the audio recordings were written in the transcripts. Participant quotations were used to illustrate the themes, but in line with assurances of anonymity given to interviewees, and no personal identifiers were used. Transcribed interviews were checked against the audio recordings for accuracy before the data were subject to thematic analysis, guided by Braun and Clarke’s [47] 6-step framework, with frequent returning to the data to sense check as we progressed through the framework. Codes were shared with the coauthors (AB, JC, and PM), and a codebook was generated. JM was aware that the thematic analysis and interpretation were influenced by previous experience and assumptions, reflexivity, subjectivity, bias, and emotion. Therefore, in line with Braun and Clarke’s advice, a self-reflexive approach was taken throughout the research, including creating a rolling memo of reflections from the time of the interviews until the end of the study. These notes were shared with the coauthors during the stages of thematic analysis.

## Results

A total of 14 participants, 10 women and 4 men, with 9 to 34 years of experience as a teacher, completed the interview.

### Overview of Findings

Teachers were unanimous in both their recognition of workplace stress and their sincere commitment to teaching. Participants revealed the physical and cultural workplace context to be one of relentless all-direction exposure, multifaceted delivery objectives, and fear of peer opinion and one in which their well-being was less important to policy makers and school heads than that of students. They described the constraining effects of the school context, which significantly affected teachers’ ability to look after their well-being. Stress management strategies, except for mindfulness in one school, were self-taught and included immediate real-time or deferred in-school practices, management practices external to the workplace as well as cumulative learning approaches designed to build coping strategies. Many teachers experienced burnout before finding successful stress self-management strategies, which included changing schools.

The pace of work meant that real-time use of health apps by teachers was rare. Outside of school, apps were used for symptom control (eg, insomnia), sometimes automatically (embedded usage that had become second nature, such as diary app entries) and frequently for relaxation and the creation of sustainable habits (such as mindfulness). Wearables were accessed by some teachers in the workplace and often used for quantification, occasionally for prompts (eg, to move or to exercise) or rewards, but wearers’ enthusiasm was rarely shared with colleagues.

There was evidence of a shifting organizational culture with the introduction of the concept of teachers’ well-being, and most staff members were both very positive about their school leadership and potentially open to leadership recommendations of supportive technology.

The findings reflect 2 overarching themes of understanding how the education workplace context constrains stress self-management and teachers’ use of digital health technology. Duration of teaching experience is shown in years after transcript quotes alongside the participant’s (P) number and sex (M or F).

### Organizing Theme A: Educational Practice Constrains Teachers’ Well-Being and Self-Management of Stress

#### Theme 1: Contextual Constraints on the Use of Technology for Stress Self-Management

Teachers described a context of work that demanded constant exposure on the front line, with their activity simultaneously driven by timetables and interrupted from all directions, which was nonstop in nature.

##### Theme 1.1: Relentless and Often Simultaneous Demands Dominate the Day

The relentless nature of the teacher’s role was illustrated by descriptions of not only delivering back-to-back lessons to class audience of up to 30 (or more) students, all with differing needs and abilities, but also having to manage junior staff members or act on their requests and respond to changeable situations. Not having any time to themselves during the day was frequently mentioned:

So as well as being a teacher I'm also head of house. So I've got the additional responsibility of that... it’s unpredictable (in) nature because you’ve got no idea what's going to happen but then obviously the time constraints of when someone has an issue and you're teaching full day it’s the time you then have to actually deal with that situation.

Quote 1, P9, F, 9 years

I don’t think a lot of people who aren’t in teaching totally get the fact. Oh, you get all these holidays and stuff. It’s like, you try teaching. You try presenting to groups of kids for five hours a day. And then getting bombarded with things that you can’t just not switch off from.

Quote 2, P4, F, 16 years

##### Theme 1.2: Social and Physical Exposure of the Teacher’s Role

The nonstop visibility of the job was an additional issue, with analogies made to acting and becoming *talked out*, having had to present and talk to people all day:

I mean I think that is one of the most exhausting things about teaching is that you are performing a role, constantly...And even if you are incredibly stressed, you have to perform a role anyway. And the responsibility and accountability on your shoulders combined with the fact that you are acting most of the day is exhausting.

Quote 3, P4, F, 16 years

There was a tangible sense of the social and physical environment dominating their sense of agency and their dignity, with no opportunity for privacy, compromising their ability to self-manage the frequent stressors.

#### Theme 2: Organizational Culture Influences Self-Management of Stress

Participants went on to describe organizational and cultural reasons for the lack of opportunity to manage stress. The school social environment was described as one in which many teachers still feared admitting the need for help, and for some, this had been exacerbated by the perception that student welfare was promoted over workforce well-being because of government policy.

##### Theme 2.1: Stigma of Not Coping Deters Help Seeking

Numerous fears were expressed on how help seeking would be perceived, with concerns including loss of job or demotion, negative peer opinion, and sense of failure. This sometimes extended to the staff not sharing helpful digital tools with peers despite occasionally recommending them to students:

No, [I haven’t recommended Mindshift to colleagues]. I think we're quite secretive by nature. I think it's such a stigma. As teachers I think we've all got a character trait that we like to do well and I think if you admit you're stressed it's like admitting you're failing.

Quote 4, P10, F, 22 years

I say it’s for research purposes, but actually it’s for me as well that I access these things. I use Pacifica.

Quote 5, P12, F, 32 years

Many participants’ concerns reflected a fear of peer perception that they might not be coping well, highlighting a culture where weaknesses were seen as having their origin in the individual rather than in the system.

##### Theme 2.2: Teachers’ Well-Being Secondary to Student Well-Being

Added to this fear of an unsympathetic social culture was the inference, from recent national policy, that the focus was solely on prioritizing student well-being, as well as the growing emphasis in the education sector on teachers meeting the mental health needs of students with little provision for their own:

You’ve really got to look after yourself because nobody else is going to look after you here. They're not because they can't...nobody’s got time to look after their staff.

Quote 6, P7, F, 16 years

So you’ve got stressed teachers trying to un-stress children which doesn’t make much sense.

Quote 7, P9, F, 9 years

Despite the rhetoric on well-being, the message that it is “ok to not be ok” reported by a few participants has not yet been normalized.

#### Theme 3: Feeling Isolated in School, but Indications That School Culture is Changing

Sometimes participants spoke about feeling isolated, but this was often implied through descriptions of trying to manage stressful times on their own. Others, notably within the smaller setting of subject departments, had been very upfront about struggles and had shared simple advice with colleagues, such as walking more slowly between lessons, or had already suggested technology solutions to stressful scenarios.

##### Theme 3.1: Individual Scenarios of Isolation

Sometimes, teachers described scenarios where stress had been overwhelming and the early warning symptoms of insomnia or nervous tics were ignored. These participants were frequently conscious of work dominating their lives, but in their isolation, they saw the ability to cope as an individual responsibility. Subsequently, their well-being suffered with occasionally devastating consequences such as nervous or marriage breakdown:

I went down to my staff base. I moved myself away from the door so nobody could look in, went to one of the last compartments, and I cried and I cried and I cried. There's nobody you can go to. When you're in this environment there's nobody. There's nobody to be able to download to. There's nobody to say are you alright. You're just completely by yourself. So the feeling of isolation...and actually...quite powerless to do things.

Quote 8, P7, F, 16 years

I used to bring three bottles of water to school and I could tell if I’d had a bad day because...I’ve had a sip out of each when I got home and I didn’t need to go to the loo. And there’d have been no space.

Quote 9, P10, F, 22 years

##### Theme 3.2: Tackling Isolation Within a Local Culture

Descriptions of subject departments were usually very warm, with heads of department describing camaraderie among colleagues, which led to a more supportive culture:

Yes, I'm known for emailing [my department] and saying use this app, I find it really useful. And I did some work looking at reducing workload and marking and being really sort of honest with staff and saying I'm really struggling with this. I struggle with workload and I want to try and make it better. So I share quite a lot.

Quote 10, P6, F, 20 years

I set up a support programme for [my colleague], based on my experience. What would really suit you? What are you happy to do? In terms of the aspects that I could control, I came to an agreement and a balance with her, just making sure to check up on her.

Quote 11, P14, F, 15 years

A majority of participants believed that the wider leadership sincerely wanted to support staff well-being, some referencing an annual staff well-being survey as well as some responses that had been acted upon.

##### Theme 3.3: Raising the Profile of Teacher Well-Being

All the staff members interviewed were or had been in a position of leadership themselves, and some were involved in their schools’ plans to raise the profile and provision of well-being for the staff. Participants were wary about gimmicks; some had noticed mental health posters appearing in their school but without an accompanying narrative. However, the suggestion that leadership could proactively recommend solutions raised genuine interest:

My job at the minute in terms of staff wellbeing and staff development is partly because there are changes that I wish to make because I've been here for a long time...I've listened to people, I've got a sense of what people say and wish to do and actually avoiding helplessness, this is something I can actually do and make that happen.

Quote 12, P11, F, 22 years

No, I would love (the Senior Leadership Team to recommend a wearable that is really good for managing stress). I like stuff like that. No, that is fine.

Quote 13, P3, F, 28 years

Despite expressions of a constricting culture in which stress management was seen as an individual and not an institutional responsibility, there was still a strong sense of trust in the leadership. Most of the staff members had worked at more than one school, and many had changed schools to find a culture in which they felt more supported or valued.

### Organizing Theme B: Some Digital Stress Technologies Can Be Used and Could Be Expanded

#### Theme 4: Stress Reduction Technologies Are Considered Useful by Teachers

Across the participants interviewed, 12 out of 14 participants already used digital tools for stress management or well-being, with 17 apps, 5 different brands of wearables, and 1 desktop program named.

##### Theme 4.1: Both the Form of Technology and Physical Workspace Influence Its Use

Smartphones were taken to school by teachers, but time, signal, and privacy constraints meant that phones remained in the teacher’s bag or coat pocket; just one teacher accessed a calming app in the privacy of her office during the day, and another accessed the Headspace app via her iPad. Over half of those interviewed used wearable technology (eg, Fitbit and smart watch) and real-time data to manage their stress and well-being:

I used [Headspace] when I was in the bath last night...just focusing on breathing and meditation. And I did it the previous night in bed, I just did like five minutes and even just after five minutes I felt quite relaxed and I thought wow, I should just do five minutes a day because it's nothing really...I think if I didn't have that app I wouldn't do it and it now sends me reminders.

Quote 14, P2, M, 17 years

Anyway, back to the Fitbit. I just love all the features of it. It’s got relax as well. Sometimes if I wake up in the middle of the night, I put the relax on. Quick, two-minute session to find a bit of calm.

Quote 15, P3, F, 28 years

##### Theme 4.2: Teachers Are Open to Schools Promoting Destressing Technologies

When asked about their response to the school leadership promoting a digital health innovation for well-being, interviewees voiced near unanimous enthusiasm and a belief that this would only happen if due diligence had been already done to conclude that such a tool was tried and trusted. They might not feel it was for them, but it could be for a colleague:

I'd look at that [stress app]. I'd look at anything because it doesn’t have to help me does it? It becomes part of a toolkit of stuff that you signpost to people...This is stuff I'm prepared to recommend. Actually this becomes our provision. This becomes what we do.

Quote 16, P11, F, 22 years

#### Theme 5: Sequelae of Stress Targeted by a Technology Intervention Informs Potential Choices

In terms of meeting their own needs, teachers managed stress using strategies that included the setting of boundaries, focusing on holistic well-being, keeping a diary, or intentional reflection on experiences. Sequelae of stress, such as elevated heart rate, shallow breathing, or racing thoughts, were often the target of conscious self-management strategies, including via digital health tools. Mindfulness was the most frequently cited stress reduction technique, both with and without digital support. Those who had wearable technology all expressed an interest in observing their patterns of behavior and activity, although they did not always welcome the attendants’ prompts. One interviewee had collated data from the wearable to successfully demonstrate to a line manager the need to change her schedule because of the potential exacerbation of a health condition. Many wearers did not share their data or advocate for their wearable despite liking the personal feedback. However, they did appreciate the data captured by technology tools, which was frequently mentioned as valuable and confirmatory.

##### Theme 5.1: Monitoring Behavior or Mood Patterns to Aid Reflection

The data gathered were used to aid reflection on reactions or responses to stress, and participants expressed the value of seeing patterns of behavior through tracking and mapping:

So it’s about patterns...My wonderful days. It's a diary [app]. And I am supposed to write down every day what I am feeling but it tends to be just what I have done that day whether it's longer or shorter or...I can look back at last year. You can see how far you've come - oh my goodness that was how I was feeling this time last year!

Quote 17, P1, M, 34 years

The only thing I’ve started, and I’m wearing it at the moment, is a heart rate monitor. That’s more for when I exercise, but I’ve been conscious that it comes up with a sleep pattern. Every morning, it’s less than the desired amount of sleep achieved, poor quality of sleep. So, it’s really interesting to see that, on a regular basis, I’m not getting the right amount of sleep.

Quote 18, P13, M, 11 years

##### Theme 5.2: Use of Technology to Directly Self-Manage Stress Sequelae

Participants used apps to access stress management techniques at home, such as dealing with anxiety or relaxing through mindfulness or meditation. Wearables were able to afford real-time intervention or information, such as helping them get back to sleep at night or bringing down their heart rate through managed breathing during a class in school:

And again, often if I feel myself getting a bit stressed [in class] I will look at my Fitbit and if I can see that my heartrate is above 80, which for me is very high, again, I will either do a breathing exercise or I'll just do something to change what I've got myself into and then just check again in a few minutes.

Quote 19, P6, F, 20 years

Currently, I like Insight Timer. I like it because I can adapt it to whatever I'm doing...At the end of the day...if I’m wound up about something, there is [a programme I can chose for example] for resentment or anxiety. It’s very, very adaptable.

Quote 20, P10, F, 22 years

##### Theme 5.3: Use of Technology for More Holistic Self-Care

Some teachers described indirect ways of reducing stress, managing their time better to impact workload or build self-efficacy. An example of this was seen through the use of the research tool TeacherTapp. This app has been designed to capture data from teachers at the end of their working day by asking them 3 research questions. By taking part, teachers themselves can review the findings and read associated articles:

It [TeacherTapp]) also links each day to some kind of educational research...and it also tells me how long it takes to read...it kind of makes me feel more professional and that kind of feeds into the stress and the workload thing doesn’t it. So although it's not a wellbeing tool I think it does work as one.

Quote 21, P6, F, 20 years

## Discussion

### Principal Findings

This study used a qualitative approach across several real-world settings to develop an understanding of the contextual influences on English midcareer high school teachers’ self-management of workplace stress and of their use of digital health tools for well-being. Consequently, we explored each in the following sections.

### Educational Practice Constrains Teachers’ Well-Being and Self-Management of Stress

The findings from the data analysis formed a detailed narrative of how self-management of workplace stress by senior teachers is subject to constraining contextual and organizational factors. Although the concept of constraints has often been cited in teaching literature [52], with workload the most cited cause of stress [53,54], details of how contextual constraints affect staff well-being or how interventions can help with stress [13] are sparse. The first of these constraints identified by participants was of relentless demands, which have been described elsewhere [55,56]. Yet, when combined with a second constraint of lack of privacy—the ability to withdraw from the front line or absence of self-care time known as *local privacy*—this absence of refuge results in inhibition, stifling of expression, or no opportunity to destress [57]. Some schools no longer provided a staff room for teachers to retreat to, yet we know from Maslach’s [58] work that stress leading to burnout is most frequent among those whose work involves intense ongoing involvement with other people, such as doctors and teachers.

Stigma of help seeking was a third constraining factor. This related to teachers’ fear of being perceived to be of *inferior ability* if they ask for help [56], as well as the stigma that continues to exist around seeking mental health support. There was a notable exception described by one participant who had a mental illness diagnosis and experienced excellent support and understanding from both the school leadership and students. However, the findings indicate that the cultural normalization of well-being support has not been achieved.

It was more often seen as ironic that the recent national policy’s emphasis on mental health in schools was almost exclusively aimed at students and not the education workforce [59], despite evidence of teachers’ concerns that if their own emotional needs are neglected they cannot meet those of the students [31,60]. These confidentiality and peer concerns mirror the opinion of health care professionals [61,62], and anonymity is still cited in research as a strong benefit to online mental health support forums [63]. Using Web-based, evidence-based therapy or psychoeducation has also been shown to reduce negative perceptions of mental illness, so there is potentially a double beneficial effect with treatment and attitude [64-66]. These factors of intensity, exposure, and apparent sidelining need to inform stress support technology design and implementation for teachers. They should also inform education policy leaders’ planning for whole-school mental health initiatives.

“Feeling lonely in a crowded room” was how one teacher described one symptom of burnout, and it was poignant how symptoms such as isolation, dehydration, hunger, and insomnia were frequently described alongside the assumption that it was purely down to the individual to manage the causes and manifestations of stress. This individualized responsibility can place a profound burden on a teacher as such an approach fails to acknowledge the complexity of the origins of stress [65]. Aspirations of the school leadership to raise the profile of teacher well-being could be harnessed through an organizational approach to address these common causes. The trust expressed in the leadership, despite the understanding that the leadership’s hands are sometimes tied by external factors such as imposed student number increases and testing, appears to be a valuable opportunity to acknowledge and consider interventions that could ameliorate stress indicators at an organizational level.

### Some Digital Stress Technologies Can Be Used and Could Be Expanded

Our second organizing theme extends previous research on the use of health technology for workplace well-being. Smartphones (and hence real-time health apps) were rarely accessed during the working day by teachers. Among the 23 mental eHealth (Web-based) at-work studies reviewed by Stratton [35] in 2017, only 3 studies seemed to utilize mobile apps, indicating that smartphones were used in a small minority of studies. Just one eHealth study was conducted among teachers, using a Web-based program. No mention was made of when the program was accessed during the day or whether it was possible to access the program via an app [41]. This study’s findings do not rule out teachers’ stress management being assisted through an app, but we can for the first time infer that apps would not be suitable for just-in-time stress relief, certainly not in the presently constructed school context.

However, the participants were nearly unanimous in their willingness to give a particular stress-relieving or prevention technology a try should the school ever promote such a technology. This could partly be a reflection of the high rate of participant use of well-being technology, which was already present in our sample (12 out of 14 participants), but it was also a clear expression of their trust in the school leadership. Most participants believed that the school leadership would only endorse a stress-relieving technology that could be valuable and reliable. This could reflect the fact that the staff members worked at schools that acknowledged staff well-being or that the staff members had chosen to work in a school where they felt valued, which engendered their trust in the leadership.

Current use of technology by teachers demonstrated the potential for combining self-management of stress and organizational promotion of well-being. However, the proportion of teachers who did (12/14, 86%) cannot be extrapolated to the whole population as they self-selected for the interview and were not a random sample. Of Nunes’ description of 5 modus operandi (MO) of technology for self-care, 2 of the 5 were found to be individually oriented: (1) fostering reflection and (2) suggesting treatment. The 3 other MO’s were communal: (3) sharing care activity, (4) enhancing collaboration, and (5) peer-to-peer support [67]. This study showed how these MO have been used in the education workplace providing valuable indicators both for potential support tools and delivery of an organizational intervention.

Among participants, methods that fostered reflection, MO (1), were the most widely used both with and without technology. In fact, 2 participants revealed how technology had become embedded, realizing during the interview that it was their apps that aided reflection. Of the 12 participants who reported using technology to support their well-being, 8 participants reported having a wearable band or watch. The real-time feedback and actionable insights of the wearables (2) were felt to be useful and empowering, enabling positive decisions to be taken to manage stress indicators. These included undertaking a breathing exercise to lower an elevated heart rate or planning more exercise. Feedback on and insight into sleep quality were mentioned by several people as provoking a change in behavior. These examples correlate with the approach of empowerment through meaningful data, facilitating participants to make changes in a way that also respects their autonomy as described by Tengland [68]. This author makes the important point that empowerment can be seen as ethically preferable to behavior change, as the latter is often concealed from the users, whereas empowerment directly informs and assists individuals, enabling better choices to be made consciously by them. Given the value placed on trust in the leadership by teachers, any organizationally directed intervention would have to be empowering and transparent.

Most teachers did not promote their wearable to colleagues, but 2 participants did mention enjoying comparing activity with other teachers as per the peer-to-peer MO (5) noted above. A recent systematic review of workplace studies utilizing mobile health, including wearable interventions to promote physical activity, has shown reasonable evidence for workplace eHealth in a workplace context as “a feasible, acceptable and effective tool” [37]. This study’s findings reveal the contextual value placed on wearables by some teachers and suggest an acceptability of this modality.

Many of the apps used facilitated immediate therapeutic access, most commonly to mindfulness, breathing, or meditative tools, combining Nunes’ (1) and (2) MO descriptions. Only one teacher claimed to use Web-based CBT, but some of the apps mentioned by the staff used CBT principles. The staff also engaged with both mindfulness and social support (eg, fit2teach) technologies. All 3 strategies aligned with the evidence for stress reduction efficacy among teachers [21], and these show self-management choices that could be of value to other senior teachers. Only the Web-based CBT resulted from a professional referral. One participant described how using the TeacherTapp app, which has been designed for education research [69], not only enhanced collaboration (the communal aspect of collaboration from Nunes’ description of MO (4) of technology for self-care) but also made her feel more professional, providing an indirect boost to her well-being. This participant also noted how forums on social media enabled peer-to-peer support. This was for professional sharing rather than direct stress management, but it still had a positive effect in terms of reducing the workload.

Most tools were adopted serendipitously, discovered through advertising, friends and family, or social media, or just occasionally through peer recommendation. Both the inclination of the staff and availability of technology suggest opportunities for technology-enhanced stress self-management that both reflect current strategies and could possibly work around the described constraints.

The UK government’s 2017 Farmer-Stevenson report included recommending public sector employers to “Ensure provision of tailored in-house mental health support and signposting to clinical help” [70]. It is hoped that the findings of this study will go some way to enabling that *tailored* support.

### Strengths and Limitations

This was a small-scale study, and the participant sample cannot be claimed to be representative of all midcareer high school teachers. In particular, as the sample was self-selecting, it is likely that there were a higher proportion of staff members who had experienced crises and time out of teaching than the average population. Beyond the confirmation of the school’s recognition of staff well-being, this study did not investigate the influence of the school’s organizational or leadership attitude on how teachers managed their stress. The participants knew in advance that they were going to be asked about their use of health technology; therefore, although 2 participants did not use any, the proportion of those who did cannot be generalized to the whole teaching population. In addition, this study was limited by a lack of ethnic or racial diversity.

All the staff members were interviewed during term time after at least five weeks back at school after the summer holidays. It was intentional that participants were interviewed while in the thick of school activity, and all of them were very much immersed in the demands of school life. Owing to their busy schedules, we are immensely grateful to the participants who gave their time, courageously talked about stressful situations, and who without exception demonstrated commitment and determination. Their pride in their students was tangible.

As a former clinician, JM was trained to look for symptoms and think diagnostically. Inevitably, although this can aid insight, it could mean a more medical bias to the analysis. However, the coauthors were from nonclinical backgrounds, and all the findings were reviewed with them.

### Conclusions

The aim of this study was to elicit the details of the contextual influences on midcareer high school teachers’ management of stress. This study’s findings are consistent with previous research among education and health care professionals on how context shapes their stress management. Distinctively, we described both the cultural and physical contextual reasons, including endless demands, lack of local privacy, stigma, inferiority, and individualism, which are constraining teachers’ workplace stress management. This study also indicated important signs or correlates of stress for teachers, including dehydration, feelings of isolation, and insomnia.

This research has provided distinct insights into how DHIs are being used for stress management and noted that mobile apps have the most potential for stress support outside the workplace, although wearable technologies can provide easy access to data within the workplace.

The important directions indicated for future teacher studies are to explore contextual changes to improve stress management, examine the correlates of stress that could be addressed by technology support, identify how and why stress symptoms can be helped using digital tools, and determine how such tools can be designed to provide enhanced, tailored support.

## Appendix

Multimedia Appendix 1 The semistructured qualitative interview guide.

## Abbreviations

CBT: cognitive behavioral therapy
DHI: digital health intervention
eHealth: electronic health
MO: modus operandi
PST: problem solving therapy
RCT: randomized controlled trial
