# Supplementary material for: How Contextual Constraints Shape Midcareer High School Teachers' Stress Management and Use of Digital Support Tools: Qualitative Study
Source: JMIR Ment Health. 2020 Apr 27;7(4):e15416. doi: 10.2196/15416 (PMC7215497; doi:10.2196/15416)
Supplement: Multimedia Appendix 1 [file mental_v7i4e15416_app1.docx]

# Appendix 1

# The Semi-structured Interview Guide

*Research Questions: The aims of this study therefore are to engage with mid-career school teachers from high (secondary comprehensive) schools to understand:*

- - *(1) how workplace context influences stress management; and*
  - *(2) how this affects current use of digital health technology.*

## Introduction notes to cover

- **Thanks:** for their time. Ensure they have the copy of the Participant Information Sheet and Consent Form. Go through consent form if not complete and ensure it is signed. Highlight the Education Support Partnership helpline contact details on the information sheet if they feel they want to talk further or receive support after this interview.
- **Topic Reminder:** This work is not looking at causes of stress, which for teachers are well documented, but the potential for effective digital health interventions (DHIs) to support teachers and reduce symptoms in the school environment.
- **Confidentiality**: The name of the participants will only be known by the researchers and not be revealed to anyone The participants will be identified by an ID and all potentially identifying information will be removed. Individual teachers and those from different schools are being interviewed and findings aggregated, so no answers can be tracked back to a particular school.
- **Participation:** Do not share information you don’t want to, there are no right or wrong answers, it is about their views – but please be as honest as they can.
- **Recording:** the session will be audio-recorded, transcribed and analysed by researchers using NVIVO software, one of the most used widely software for qualitative analysis.

## Questions

1. Which years do you teach/ how long have you been a teacher / why did you choose to teach (your subject)?
2. How often do you feel stressed at work, and is it a brief feeling or something more long-lasting and pervasive?
3. Lots of research has been done of the causes of stress, and if you think back to an instance recently that you felt was stressful, can you describe how you dealt with it – either in school or when you got home?
4. Is that how you normally manage stress / stressful situations? Does that approach help – and why?
5. Work on stress symptoms has divided them into four groups, can I ask you about whether you have experienced any of them? This isn’t a diagnosis, it is just to get a sense of the most common symptoms amongst teachers – yes/no.

Emotional changes

1. irritability or agitation
2. general unhappiness
3. loss of sense of humour
4. poor self-esteem
5. feeling demotivated
6. becoming withdrawn
7. feeling isolated

Thought changes

1. impaired judgement
2. finding it hard to concentrate
3. Indecision
4. racing thoughts or mind blanks (in the moment)

Behavioural changes

1. floor pacing
2. nervous habits
3. increased sickness absence

Physical changes

1. aches and pains
2. fatigue
3. sweating
4. headaches
5. shallow breathing
6. accident prone
7. stomach upsets
8. fast heart rate
9. disregard for personal appearance
10. Do you think your school acknowledges staff wellbeing and stress? What do they do?
11. Has the school ever taught any stress management techniques?

Prompt:

- Diaphragm breathing / PMR
- Meditation / mindfulness
- Cognitive restructuring
- Active coping / behavioural activation
- Seeking social supports
- Problem solving

1. Have you shared any thoughts on stress or exchanged ideas with work colleagues? Sharing stuff/resources in a timely way.

Why / why not (Barriers and openings).

1. You are probably aware of apps, wearables, online programmes for health and wellbeing. Do you yourself use or have ever used any digital health /wellbeing devices e.g. fitbit, wellbeing app etc? (separate out wellbeing from chronic condition e.g. diabetes, asthma).

If yes, prompts:

- - What do you like about the DHI?
  - Do you use it in school?
  - Did you have to make any adjustments to be able to use it in school?
  - How do you feel about students/staff knowing you use this tech?
  - Do you think other staff members would be interested in using the same tech?
  - Is there anything that makes it especially suited to a school environment/ culture/context?
  - If so, what factors could prevent this?

1. Have you used a DHI to reduce stress?

If yes, what?

If not – any reason why not?

What do you think would be helpful / effective?

Why wouldn’t you use one?

1. How would they feel about the school promoting a particular DHI?
2. What was the last moment in your teaching career when you thought yes - this is all worth it?!
